# Ultrafast Thermionic Electron Injection Effects on Exciton Formation Dynamics at a van der Waals Semiconductor/Metal Interface

**DOI:** 10.1021/acsphotonics.2c00394

**Published:** 2022-07-20

**Authors:** Kilian R. Keller, Ricardo Rojas-Aedo, Huiqin Zhang, Pirmin Schweizer, Jonas Allerbeck, Daniele Brida, Deep Jariwala, Nicolò Maccaferri

**Affiliations:** † Department of Physics and Materials Science, University of Luxembourg, 162a Avenue de la Faïencerie, L-1511 Luxembourg, Luxembourg; ‡ Department of Electrical and Systems Engineering, University of Pennsylvania, 19104 Philadelphia, Pennsylvania, United States; § Nanotech@Surfaces Laboratory, EMPA, Ueberlandstrasse 129, 8600 Dübendorf, Switzerland; ∥ Department of Physics, Umeå University, Linnaeus väg 24, SE-90187 Umeå, Sweden

**Keywords:** exciton dynamics, thermionic electron injection, metal−semiconductor heterojunction, transition metal dichalcogenides, ultrafast dynamics, hot-electrons

## Abstract

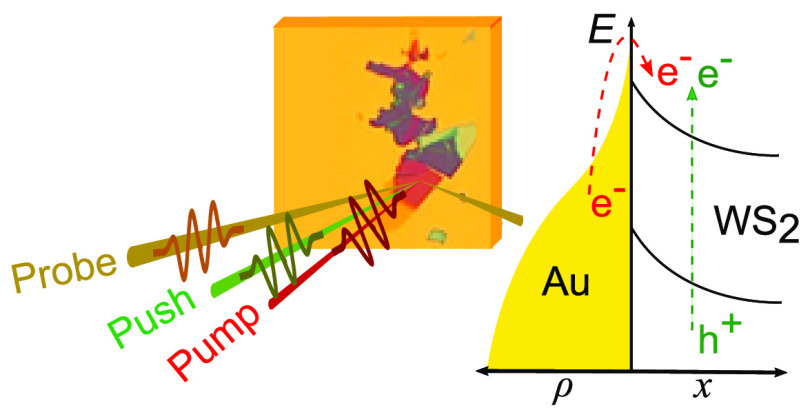

Inorganic van der Waals bonded semiconductors such as transition metal dichalcogenides are the subject of intense research due to their electronic and optical properties which are promising for next-generation optoelectronic devices. In this context, understanding the carrier dynamics, as well as charge and energy transfer at the interface between metallic contacts and semiconductors, is crucial and yet quite unexplored. Here, we present an experimental study to measure the effect of mutual interaction between thermionically injected and directly excited carriers on the exciton formation dynamics in bulk WS_2_. By employing a pump–push–probe scheme, where a pump pulse induces thermionic injection of electrons from a gold substrate into the conduction band of the semiconductor, and another delayed push pulse that excites direct transitions in the WS_2_, we can isolate the two processes experimentally and thus correlate the mutual interaction with its effect on the ultrafast dynamics in WS_2_. The fast decay time constants extracted from the experiments show a decrease with an increasing ratio between the injected and directly excited charge carriers, thus disclosing the impact of thermionic electron injection on the exciton formation dynamics. Our findings might offer a new vibrant direction for the integration of photonics and electronics, especially in active and photodetection devices, and, more in general, in upcoming all-optical nanotechnologies.

## Introduction

Heterojunctions of metals and semiconducting transition metal dichalcogenides (TMDs) allow various possibilities for the manipulation and exploitation of light–matter interactions, such as the control of plasmonic excitations^1−4^ and plasmon-induced charge injection,^5−9^ transistors,^10^ and photovoltaics.^11^ Due to their layered structure, excited electrons and holes in TMDs exhibit enhanced Coulomb interactions in both monolayer and bulk (>5 layers) forms,^12^ leading to room-temperature stable excitons, which dominate the optical and charge transport properties in these materials. Furthermore, TMDs form atomically clean and sharp interfaces with other materials,^13^ which makes them ideal candidates for optoelectronic applications where high-quality interfaces between metals and semiconductors are essential. Moreover, TMDs potentially offer a superior alternative to other semiconductors, as TMD/metal interfaces show weak Fermi-level pinning.^14^ For these reasons, the exploitation of TMDs for opto-electronics is currently the subject of intense research^15^ where different degrees of freedom, such as manipulation of the dielectric environment^16^ and exciton–plasmon interaction,^9^ have been explored. As well, the ultrafast electronic dynamics of isolated 2D and bulk TMDs interfaced with insulating substrates have been the focus of recent studies.^17−19^

Here, we show a new perspective to study the interplay between carrier injection and exciton formation dynamics at a van der Waals semiconductor/metal interface in view of future applications which exploits the ultrafast (sub-ps) opto-electronic properties of TMDs. In more detail, we focus on how the ratio between thermionically injected and directly excited charge carriers affects the exciton formation dynamics in a bulk TMD/metal heterojunction. It has been shown theoretically that an excess of free electrons in the conduction band of TMDs compared to the density of free holes affects the probability to form neutral and charged excitons, that is, trions.^20^ Also, experiments showing that an excess of electrons in the conduction band due to n-doping can modulate the excitonic absorption have been reported.^21^ Furthermore, recent studies reveal that at WS_2_/semimetal heterojunctions, hot carriers injected from the semimetal into a TMD are able to affect the exciton formation dynamics by comparing the transient signal of pump–probe experiments for pumping above and below the optical band gap of the TMD.^22−24^ We designed an experiment to pump below and above band gap in parallel which allows us to extract the effect of mutual interaction between injected and excited charge carriers on the transient signal in the absorption line of the exciton. To be specific, we measure the ultrafast transient response of the heterojunction employing a three-pulse pump–push–probe (PPP) configuration, which is motivated by a standard pump–probe (PP) with pump energy above and below the optical band gap. The PPP enables us to disentangle the effect of hot-electron injection from the metallic substrate from the direct excitations in the semiconductor.

## Results and Discussion

The TMD employed in our study is tungsten disulfide (WS_2_), a promising material for applications given its superior charge transport performance compared to other TMDs^25^ and, most importantly, because it displays a single and very strong primary exciton feature which dominates the optical spectrum even in the bulk form and at room temperature. The A-exciton exhibits a binding energy of about 50 meV given an electronic band gap at the *K*-point of 2.1 eV^12,26^ in bulk WS_2_. We also chose a bulk sample of WS_2_ instead of the monolayer due to higher absorption and lower contact resistance at the TMD/metal interface for charge injection.^16^ After optical excitation, the ultrafast dynamics in inorganic semiconductors are dominated by carrier–carrier (c–c) scattering, that involves electron–electron and electron–hole scattering, promoting exciton formation, which typically happens on a timescale less than 1 ps.^19^ Therefore, by probing our system at the absorption line of the A-exciton the measured transient signal in this time frame is a fingerprint of the A-exciton formation dynamics affected by free charge carriers, that is, via c–c scattering. In our study, we observe these sub-one ps dynamics by exciting a free electron–hole plasma in the WS_2_ with a light pulse tuned to an energy above the band gap at the *K*-point, and narrowband enough to ensure that the pulse does not directly excite the A-exciton. For the metal, we employed gold because it displays a large work function (WF) of approximately 5.1 eV, thus leading to a lower Fermi level pinning effect and oxidation that otherwise would introduce additional resistance for injection.^27^ The fact that gold has a WF that exceeds the electron affinity of WS_2_ is of further importance because, in the reverse case, an accumulation layer for electrons would form at the WS_2_/Au interface with a built-in field that impairs the injection of electrons into the semiconductor. To draw conclusions about the effect of the charge injection on the ultrafast electronic dynamics in the WS_2_, we also measured as reference a WS_2_ sample deposited on a SiO_2_ substrate.

**Figure 1:**
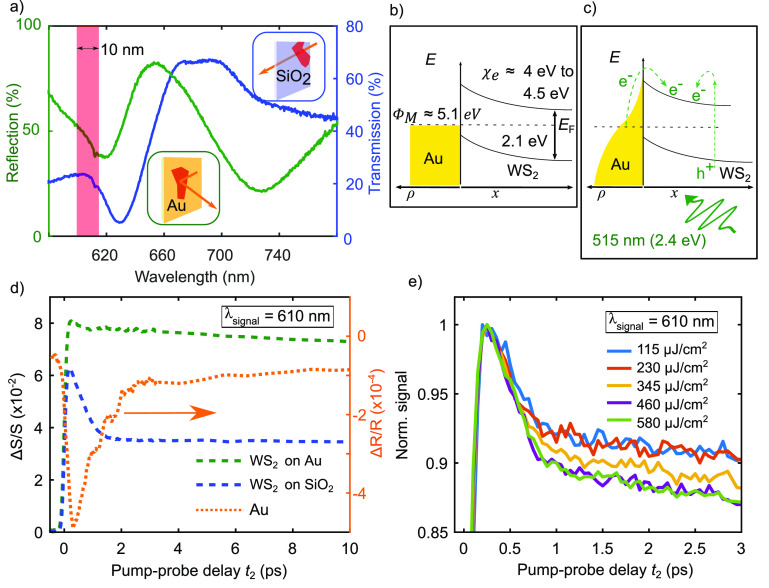
PP transient absorption measurement on WS_2_/Au and WS_2_/SiO_2_ at λ_signal_ = 610 nm (2.03 eV). (a) Steady-state spectra in reflection of WS_2_/Au (green) and in transmission of WS_2_/SiO_2_ (blue). Red bar indicates the spectral width of band pass centered at 610 nm (2.03 eV). (b) Thermal Fermi–Dirac distribution (ρ) in gold and band alignment in WS_2_ for WS_2_/Au heterojunction with approximate values for gold WF Φ_M_, electron affinity χ_e_ of WS_2_, and indication of Fermi energy level *E*_F_. (c) ρ of gold and band alignment in the case of illumination by a light pulse with indication of a direct excitation of free electrons (e^–^) and holes (h^+^) and thermionically injected electrons. (d) PP measurements on WS_2_/Au (green dashed line), WS_2_/SiO_2_ (blue dashed line), and bare gold substrate (orange dotted line). Pump at 515 nm (2.4 eV) with fluence of 200 μJ/cm^2^ and visible white light probe with a fluence of 40 μJ/cm^2^. Δ*S*/*S* represents either transient reflection Δ*R*/*R* for WS_2_/Au or transient transmission Δ*T*/*T* for WS_2_/SiO_2_. (e) Normalized Δ*R*/*R* PP measurement of WS_2_/Au for different pump fluences.

Figure 1a shows the steady-state spectra of WS_2_/Au (green) and WS_2_/SiO_2_ (blue) in reflection and transmission, respectively. The dips at 618 nm (2.01 eV) for WS_2_/Au and at 630 nm (1.97 eV) for WS_2_/SiO_2_ correspond to the absorption of the A-exciton. The slight difference in the excitonic resonances can be attributed to a different screening from the gold at the WS_2_/Au interface compared to the WS_2_/SiO_2_ sample.^28^ From the spectral position of the etalon mode at 730 nm, (1.70 eV) we can determine the thickness of the WS_2_ flake on gold,^4^ which is about 20 nm. The WS_2_/SiO_2_ sample has an approximate thickness of around 100 nm. The difference in thickness does not affect the ultrafast electronic response in WS_2_ because both can be considered to be bulk.^17^ In the PP measurements, we focus on the neutral A-exciton absorption spectral region, which has an additional minor contribution from the negatively charged trion absorption at slightly lower energy with respect to the A-exciton. For this reason, we detect the probe signal by using a band-pass filter centered at 610 nm (2.03 eV) with a spectral width of 10 nm, depicted by the red bar in Figure 1a.

**Figure 2:**
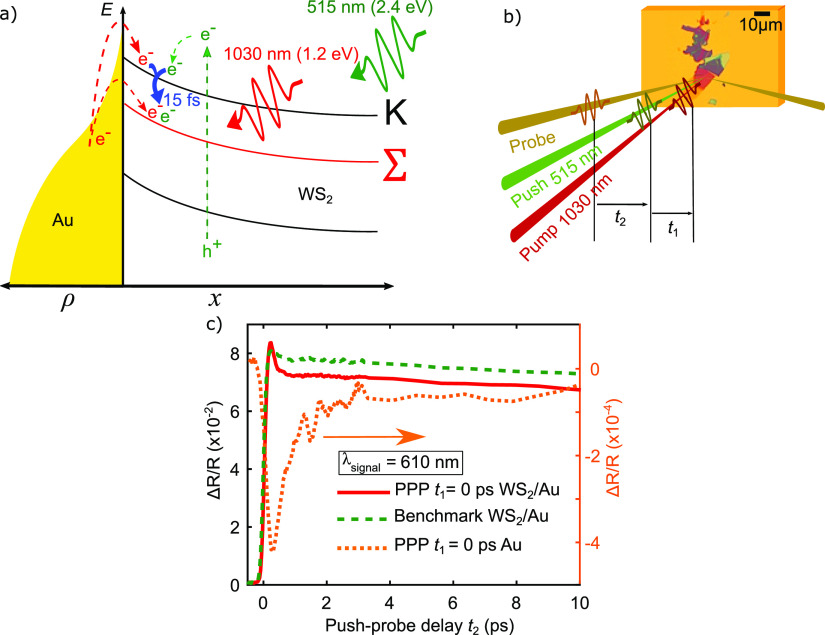
PPP experiment on WS_2_/Au at λ_signal_ = 610 nm (2.03 eV). (a) Thermal Fermi–Dirac distribution (ρ) in gold and band alignment in WS_2_ for the WS_2_/Au heterojunction under illumination by pump pulse (red), followed by a modulated push pulse (green). The pump-induced thermionic injection of excess electrons (e^–^) from the gold and the direct excitation of free electrons (e^–^) and holes (h^+^) by the push pulse in WS_2_ is indicated by dashed arrows. The blue arrow indicates intervalley scattering, which migrates excited and injected electrons from the *K* to the Σ valley on a time scale of 15 fs.^34^ (b) PPP configuration and microscopy image of WS_2_ flakes on gold with the indication of fixed pump–push delay *t*_1_, scanned push–probe delay *t*_2_, and central wavelengths. (c) PPP measurement with pump–push delay *t*_1_ = 0 ps on WS_2_/Au (red line), bare gold (orange dotted line) and reference PP measurement on WS_2_/Au (green dashed line).

The WS_2_ sample is directly exfoliated on gold (Supporting Information—Note 5), leading to weak electronic coupling, which results in the formation of a Schottky junction^29^ with the distribution of metal electronic states and band bending in the WS_2_^27^ in proximity of the interface as sketched in Figure 1b. In our case, an important parameter affecting the contact resistance is the so called Schottky barrier height (SBH), which is the potential barrier that the hot electrons have to overcome in order to be injected from the gold into the conduction band of the WS_2_. For the WS_2_/Au junction, the SBH is approximately 1 eV.^27^ In the PP study on WS_2_/Au and WS_2_/SiO_2_, we use an optical pulse centered at 515 nm (2.4 eV) with a pulse duration of about 150 fs, a bandwidth of 5 THz, and a fluence of 200 μJ/cm^2^ to pump the system. This pump fluence excites an electron–hole density on the order of 10^13^ cm^–2^ on the surface layer of the WS_2_ sample, which is 2 orders of magnitude higher than in other studies on ultrafast dynamics in TMDs.^17−19^ Since the aim of this work is to study the effect of injected charge carriers on exciton formation, a large cross section in between the injected and directly excited charge carriers can be realized with a high density of excited carriers. This excitation density has been chosen because it is below the regime where the exciton would be ionized due to band gap renormalization, where the transient vanishing of the excitonic resonance in the range of few hundreds of femtosecond after excitation is identified as a hallmark of this regime.^30^ The measured transient spectra on WS_2_/Au for pump–probe delays (*t*_2_) in the range of 0.2–0.8 ps (Figure S1a in the Supporting Information) shows that the absorption associated with the A-exciton does not disappear for a fluence of 200 μJ/cm^2^, which implies that the transition from an excitonic to a fully plasma dominated regime does not take place in our case. As a probe pulse, we employ a visible supercontinuum with a fluence of about 40 μJ/cm^2^. We refer to this first PP study as our benchmark measurement throughout the paper. Figure 1c depicts the conditions of this first experiment in which we can observe two main effects due to the pump pulse: (i) an increase of the electronic gas temperature in gold facilitates thermionic injection of hot electrons into the conduction band of WS_2_, and (ii) a direct excitation of free electrons and holes in the WS_2_. Figure 1d shows the transient response (Δ*S*/*S*) of WS_2_/Au (green dashed line), WS_2_/SiO_2_ (blue dashed line), and bare gold (orange dotted line) as a function of PP delay *t*_2_. The measurements show that the presence of a Schottky interface causes fundamentally different dynamics. In order to extract a time constant for the fast decay a single exponential model (see Supporting Information—Note 6) is used. The extracted fast decay time constants of the benchmark curves are smaller for WS_2_/Au compared to WS_2_/SiO_2_ with τ_B,WS_2_/Au_ = 324 ± 7 fs and τ_B,WS_2_/SiO_2__ = 596 ± 8 fs, respectively. A relevant observation here is that the measured dynamics of WS_2_/SiO_2_ agree with previous results on similar samples.^18^ The difference between the curves cannot be explained just through the transient response of the gold because the signal from the bare gold alone is 2 orders of magnitudes lower. Therefore, it is likely that this change in dynamics is related to the free charges injected from the metal into the semiconductor, which via c–c scattering, screening, and renormalization, in combination with the screening effect from the semiconductor/metal interface, can modify both the SBH and the exciton binding energy. It is worth mentioning that the effect from the injection and the interface effects, that is, screening of the WS_2_ by the gold, are not strictly separable, as the injection changes the density of free electrons in the metal and that in turn modifies the interface itself. From Figure 1d, we observe that there is a strong change of the dynamics for *t*_2_ < 1 ps in the two cases. This temporal regime is mainly dominated by c–c scattering,^19^ which includes scattering between injected and excited charges and a non-negligible contribution from carrier–phonon (c–ph) scattering^31^ in the WS_2_. We would therefore expect a change of dynamics at this timescale upon changes of the density of carriers either excited or injected. Therefore, we varied the fluence of the pump pulse and measured the transient absorption on the WS_2_/Au. The normalized signals in Figure 1e show that the dynamics for *t*_2_ < 1 ps are not changing significantly upon variation of the pump fluence. For larger delays *t*_2_, there is an offset for different fluences, which can be attributed to a temperature dependence of the excitonic resonances.^32^ To understand the results for *t*_2_ < 1 ps, one first step is to remember the two main effects caused by the pump pulse. In the range in which the pump fluence is varied the density of injected and the directly excited carriers in WS_2_ are simultaneously modified proportionally. In the case of a strong interaction between the injected electrons and the charge carriers in WS_2_, it is reasonable to expect a significant change in the excited carrier dynamics especially when the ratio between the density of injected and excited carriers is varied. As shown theoretically in ref (20), the probability to form excitons in TMDs is modulated as a result of varying the density ratio between electrons in the conduction band and holes in the valence band. Therefore, we expect a modulation of the ultrafast dynamics in the absorption line of the A-exciton by varying this carrier ratio. For this reason, it is necessary to control the injection independently from the excitation. More importantly, in order to understand how an ultrafast excitation in the WS_2_ responds to an injection of electrons from the gold, the two processes have to be separated. This means that the thermionic injection from the gold and the carrier excitation in WS_2_ should be generated by two different and independent light pulses. For this reason, we performed a second study where we implemented a three-pulse PPP measurement scheme to detect the transient response of our heterojunction. Figure 2a depicts the outline of the PPP experiment. We refer to the first pulse arriving at the interface as “pump”, using the fundamental wavelength (FW) of the laser amplifier at 1030 nm (1.2 eV), with a fluence of 1.7 mJ/cm^2^ and a temporal duration of 220 fs.

The purpose of this first pulse is to increase the electronic temperature of gold and promote the thermionic injection of electrons into the WS_2_. While it is true that the injection process requires the energy of the injected electrons to be higher or very close to the SBH, the energy of the photons exciting the metal electron gas is not the main parameter affecting the injection density. This partial independence with respect to the photon energy is mainly due to a thermalization from an initial non-thermal distribution in the first tens of femtoseconds in Au,^33^ driven mainly by e–e scattering. This implies that while in the first femtoseconds the injection may be dependent on the pump photon energy, after tens of femtoseconds the out-of-equilibrium distribution is no longer relevant and the temperature of the electron gas is the main origin of high-energy electrons. On the other hand, with a photon energy of 1.2 eV, this pulse cannot directly excite carriers in the semiconductor. Furthermore, we do not observe an ionization of the A-exciton due to thermionically injected electrons as shown in Figure S1b (Supporting Information). Also, the contribution of two-photon absorption is negligible because the signal scales linearly with the fluence ranging from 0.8 to 7.2 mJ/cm^2^, as shown in Figure S2 (Supporting Information). The subsequent pulse is called “push”, and it is the previously used second harmonic of the FW at 515 nm (2.4 eV) with a fluence of 200 μJ/cm^2^. Thus, it has sufficient photon energy to excite an electron–hole plasma in the WS_2_. The system is then probed in the same way as in the PP experiments. The introduction of an additional pump pulse with photon energy below the electronic band gap of the WS_2_ is the essential part of our work as it injects excess charges that allow us to largely separate the hot-electron injection from the gold from the carrier excitation in the WS_2_. Furthermore, PPP enables us to change the ratio between injected and excited charges by changing the fluence or by adding a temporal delay between the pump and the push pulses. It is important to note that changes of this ratio imply that we can explore a different environment for c–c scattering in WS_2_ which would affect the dynamics of processes occurring on the timescale <1 ps, for example, exciton formation. It is worth mentioning here that in the case where a delay in between pump and push pulses is introduced, dynamics such as intra-band population migration mechanisms should be considered in the analysis because they alter the ratio of injected and excited charges in the probed excitonic absorption band at the *K*-point during this delay time. Bulk WS_2_ exhibits a fast intervalley *K*–Σ scattering on a timescale of 15 fs^34^ as indicated by the blue arrow in Figure 2a, which results in a migration of electrons from the local conduction band minimum at the *K*-point to the global conduction band minimum at the Σ-point. Compared to the more conventional PP scheme, the PPP configuration enables to study carrier dynamics in the WS_2_ system already in contact with “hot” gold or, in other words, with a hot-electron reservoir. Figure 2b summarizes the PPP measurement scheme, in which the pump pulse arrives at a fixed delay *t*_1_ before the modulated push pulse, which is followed by the probe pulse with variable delay *t*_2_. Additionally, heating of the gold due to the push pulse can be neglected because the fluence of the pump pulse is about an order of magnitude higher and the injection across the Schottky barrier scales nonlinearly with the fluence. In Figure 2c, we plot the result of a PPP measurement on WS_2_/Au (red line) and on the bare gold substrate (orange dotted line), for the case *t*_1_ = 0 ps, that is, when pump and push pulse arrive at the same time, and the benchmark measurement (green dashed line). By comparing the PPP on WS_2_/Au with the benchmark measurement, it is evident that by adding the pump pulse the dynamics for *t*_2_ < 1 ps are qualitatively different, as the time constant associated with the fast decay component seems to become shorter in the PPP configuration. The fact that c–c scattering is the dominant effect for short delays *t*_2_ implies that the pump in the PPP configuration introduces a different environment for scattering in the WS_2_ by altering the injected to excited carrier ratio. As in the case of the PP study, we can see from the PPP measurements on the bare gold that the contribution from the metal alone is 2 orders of magnitude weaker and thus cannot explain this difference in dynamics.

**Figure 3:**
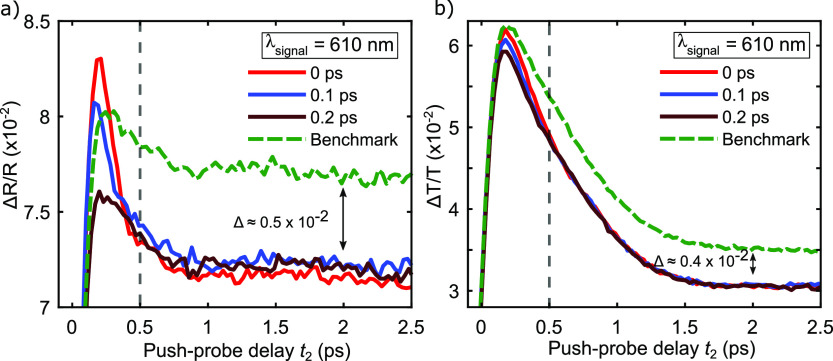
PPP on (a) WS_2_/Au and (b) WS_2_/SiO_2_ at λ_signal_ = 610 nm (2.03 eV) for different pump–push delays *t*_1_ and PP reference (green dashed line). The gray dashed line indicates the delay *t*_2_ from which on different *t*_1_ curves follow the same dynamics.

To better understand the change of dynamics caused by the hot-electron injection due to the pump pulse, we performed PPP measurements for different pump–push delays (*t*_1_) on the WS_2_/Au sample, and also compared the results with those observed in the case of the WS_2_/SiO_2_ reference sample. Figure 3a shows the PPP measurement at 610 nm (2.03 eV) on the WS_2_/Au sample for the cases when the pump arrives 0 ps (red line), 0.1 ps (blue line), and 0.2 ps (brown line) before the push pulse.

The PPP curves show qualitatively strong variation in dynamics for delays *t*_2_ < 0.5 ps (highlighted by the gray dashed line). For longer delays *t*_2_, the curves follow the same dynamics with a constant offset with respect to the benchmark (green dashed line) of about Δ*R*/*R* ≈ 0.5 × 10^–2^. Similar to the discussion of Figures 1d and 2c, two temporal regimes must be distinguished, with the difference that in PPP an additional contribution to c-c scattering with the excess carriers for short delays *t*_2_ < 0.5 ps is introduced. In this first regime, the PPP measurement for *t*_1_ = 0 ps shows significantly different dynamics with respect to the benchmark, featuring a faster decay after the maximum of the Δ*R*/*R* signal. With increasing pump–push delay *t*_1_, the dynamics approach the benchmark case recovering the same fast decay value for *t*_1_ = 0.2 ps. In Figure 3b, we show the results of the PPP measurement at 610 nm (2.03 eV) on the WS_2_/SiO_2_ reference sample for different *t*_1_. Similar to the WS_2_/Au sample, the experimental curves display different dynamics in regime I (*t*_2_ < 0.5 ps) (gray dashed line), which for long *t*_2_ delays converge and exhibit a comparable offset of Δ*T*/*T* ≈ 0.4 × 10^–2^ with respect to the benchmark, similar to that observed in the WS_2_/Au case. This similarity in regime II (*t*_2_ > 0.5 ps) is reasonable, as the charge injection from the gold substrate is mostly affecting the short *t*_2_ delays, and the effect of the pump in PPP for longer *t*_2_ delays, that is, heating of the system to different equilibrium temperatures, is similar for WS_2_/Au and WS_2_/SiO_2_.

In the first regime, the variation of the dynamics upon changing *t*_1_ for WS_2_/SiO_2_ seems qualitatively smaller with respect to the dynamics in WS_2_/Au. This observation can be verified by comparing the extracted time constants for *t*_1_ = 0 ps which are  = 157 ± 2 fs and , = 521 ± 5 fs and for *t*_1_ = 0.2 ps which are  = 283 ± 9 fs and  566 ± 7 fs with the time constants of the benchmark curves τ_B,WS_2_/Au_ = 324 ± 7 fs and τ_B,WS_2_/SiO_2__ = 596 ± 8 fs. The change of the dynamics can be quantified by introducing a percental relative variation of the time constants [Δ_τ,i_(*t*_1_)] defined as Δ_τ,i_(*t*_1_) = [τ_B,i_ – τ_i_^′^(*t*_1_)]/τ_B,i_ × 100, with i = WS_2_/Au, WS_2_/SiO_2_. This yields a Δ_τ,i_(*t*_1_ = 0 ps) of approximately 50% and 10% for WS_2_/Au and WS_2_/SiO_2_, respectively, and for Δ_τ,i_(*t*_1_ = 0.2 ps) 13% in the case of WS_2_/Au and 5% in the case of WS_2_/SiO_2_. The observation that Δ_τ,WS_2_/SiO_2__(*t*_1_) does not go to zero for *t*_1_ = 0 ps and *t*_1_ = 0.2 ps is ascribed to a different thermal state of the heterojunction at the moment of excitation. The fact that Δ_τ,i_(*t*_1_ = 0 ps) is 5 times larger in WS_2_/Au strongly supports the impact of charge injection across the Schottky interface on the absorption band of the A-exciton.

The measured net effect of injected electron density in the *K*-valley at the time of excitation by the push pulse depends on *t*_1_, lifetime of the excited charges in the *K*-valley, and the time resolution of the experiment. For our case, where the *K*–Σ migration time of 15 fs^34^ is much shorter than our temporal resolution, we assume that the net effect of the injected electron density is directly related to the instantaneous electronic thermal distribution at the time *t*_1_. By changing the delay *t*_1_, the instantaneous thermal distribution of electrons in gold is different at the moment of excitation in WS_2_ induced by the push pulse. Therefore, the fact that the ratio between Δ_τ,WS_2_/Au_(*t*_1_ = 0 ps) and Δ_τ,WS_2_/SiO_2__(*t*_1_ = 0 ps) is reduced from five to two upon increasing *t*_1_ by 200 fs implies a dependence on the thermal electronic distribution in gold, which generates a higher rate of change of the dynamical constants in the WS_2_/Au case.

**Figure 4:**
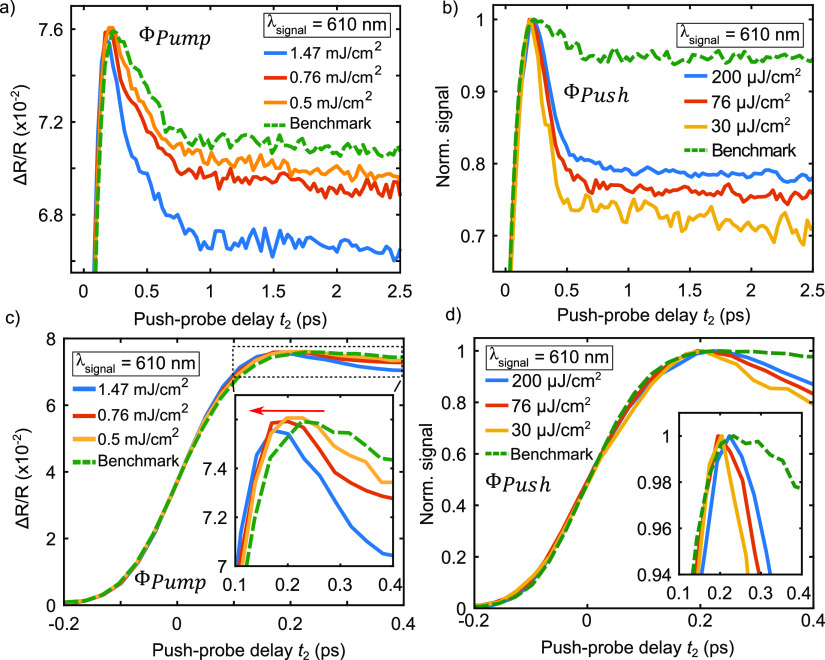
PPP curves at fixed pump–push delay *t*_1_ = 0.1 ps at λ_signal_ = 610 nm (2.03 eV) on WS_2_/Au for different pump (Φ_pump_) (a,c) and push (Φ_push_) (b,d) fluences. Lower panels show the build-up dynamics of the measurements in (a,b). The inset in (c,d) highlights the dynamics during the rise time with red arrow indicating the steepening of rise dynamics with increasing pump fluence.

To investigate how the hot electrons affect the dynamics which take place within the time scale for *t*_1_ < 0.5 ps, in the subsequent PPP measurements, we varied either the push or the pump fluences. We focused on the fixed pump–push delay of *t*_1_ = 0.1 ps to avoid undesired effects at *t*_1_ = 0 ps due to the temporal overlap of pump and push pulse, that is, coherent artifacts. Figure 4a shows the Δ*R*/*R* signal at 610 nm (2.03 eV) obtained on WS_2_/Au with a fixed push fluence of 200 μJ/cm^2^. The curves show a systematic decrease of the time constant for the fast decay from 309 ± 4 to 252 ± 4 fs with the increase of the pump fluence from 0.5 to 1.47 mJ/cm^2^. This result strongly suggests an impact of the ratio between the excited and injected charges. Since the pump is only contributing to the injection of charges, the increase of the pump fluence, given a constant density of excited charges, increases the ratio *R*_n_ = *n*_injected_/*n*_excited_. Therefore, an increase of *R*_n_ can be related to a decrease of the time constant.

To further explore the role of *R*_n_, we varied the fluence of the push pulse centered at 515 nm, which, in a first approximation, is only contributing to the excitation of charge carriers. Figure 4b shows the normalized Δ*R*/*R* signal at 610 nm (2.03 eV) obtained on WS_2_/Au for push fluences ranging from 30 μJ/cm^2^ (yellow line) to 200 μJ/cm^2^ (blue line) with a fixed pump fluence of 1.7 mJ/cm^2^ and the benchmark experiment (green dashed line). The curves show a systematic increase of the time constant for the fast decay from 147 ± 4 to 184 ± 3 fs with increasing push fluence. As the fluence of the push pulse increases, *R*_n_ decreases, and the fast decay time constant tends to the benchmark value τ_B,WS_2_/Au_. One way to understand this trend is to assume the limiting case where the excited charges outweigh the contribution of the injected charges, *R*_n_ tends to zero, in which case the time constant of the benchmark experiment must be recovered.

An additional qualitative observation can be made with respect to the build-up dynamics of the curves shown in the Figure 4a,b. Qualitatively, the rise time seems to become shorter for higher pump fluences (Figure 4c). On the other hand, the push pulse fluence does not seem to impact the rise time (Figure 4d), which implies that the dependence of the rise time on the pump fluence is not related to the ratio *R*_n_ but instead to the density of injected electrons. Finally, many body effects that cause a blue or red shift of the excitonic resonance can be considered unlikely because the maximum amplitude of the differential signal does not vary for different pump fluences.

## Conclusions

We explored via PPP how thermionic electron injection at a WS_2_/Au interface affects the transient signal associated with the A-exciton formation dynamics in the semiconductor. Different dynamics are observed in WS_2_ by varying the ratio *R*_n_ between the electrons injected from the gold and the charge carriers excited in WS_2_. This approach enables the possibility to actively modulate the fast decay time in this type of systems. We showed that *R*_n_ can be varied in three ways: (i) by changing the time delay between pump and push pulses *t*_1_, (ii) by directly varying the pump fluence while keeping the push fluence constant, or (iii) by changing the push fluence at constant pump fluence. The first two approaches are equivalent because both are changing the instantaneous electronic thermal distribution in the gold at the time of excitation. Our results show that the time constant of the fast decay decreases with increasing *R*_n_. The effect of excess carriers induces a change in the rate of c–c scattering in WS_2_ and consequently modifies the screening of the dielectric environment and the probability to form charged excitons, that is, trions, affecting intrinsically the exciton formation dynamics. One effect that was observed in the PPP that can be attributed solely to the effect of charge injection is a qualitative tendency for the rise time to decrease as the pump fluence increases, while the maximum transient signal amplitude remains unchanged. This effect, which turns out to be independent of the ratio between injected and excited charges, cannot be attributed to many-body effects that generate a blue shift or red shift of the excitonic resonance and to the best of our knowledge has not been predicted by any theoretical model. Our findings introduce an alternative approach to couple optoelectronic properties of a TMD/metal, or more general a van der Waals semiconductor/metal interface, as well as how to affect exciton dynamics through electron injection across the Schottky barrier induced by an ultrashort optical pulse. Thus, we foresee a potential impact of our results on research fields that target the exploitation of ultrafast phenomena at the boundary of photonics and electronics.

## Supporting Information Available

Measurements on the WS_2_/Au sample: transmission spectra in PP and PPP configurations, and 1030 nm pump pulse fluence dependence pump–probe measurements; AFM measurement of the bare gold substrate; additional details on the experiments, sample fabrication, and fitting procedures (PDF)
