# Supplementary material for: Ultrafast Thermionic Electron Injection Effects on Exciton Formation Dynamics at a van der Waals Semiconductor/Metal Interface
Source: ACS Photonics. 2022 Jul 20;9(8):2683–90. doi: 10.1021/acsphotonics.2c00394 (PMC9389617; doi:10.1021/acsphotonics.2c00394)
Supplement: Supplementary file 1 — ph2c00394_si_001.pdf [file ph2c00394_si_001.pdf]

Supplementary Information

Ultrafast Thermionic Electron Injection Effects on Exciton Formation Dynamics at a van der Waals Semiconductor/Metal Interface

Kilian R. Keller[1¶], Ricardo Rojas-Aedo[1¶], Huiqin Zhang[2], Pirmin Schweizer[1], Jonas Allerbeck[1,3], Daniele Brida[1], Deep Jariwala[2], and Nicolò Maccaferri[1,4*]

1. Department of Physics and Materials Science, University of Luxembourg, 162a avenue de la Faïencerie, L-1511 Luxembourg, Luxembourg

2. Department of Electrical and Systems Engineering, University of Pennsylvania, Philadelphia, PA, 19104, USA

3. Nanotech@surfaces Laboratory, EMPA, Ueberlandstrasse 129, 8600 Dübendorf, Switzerland

4. Department of Physics, Umeå University, Linnaeus väg 24, SE-90187 Umeå, Sweden

[¶]These authors contributed equally to this work

[*]nicolo.maccaferri@umu.se

Supplementary Note 1: Transient spectra

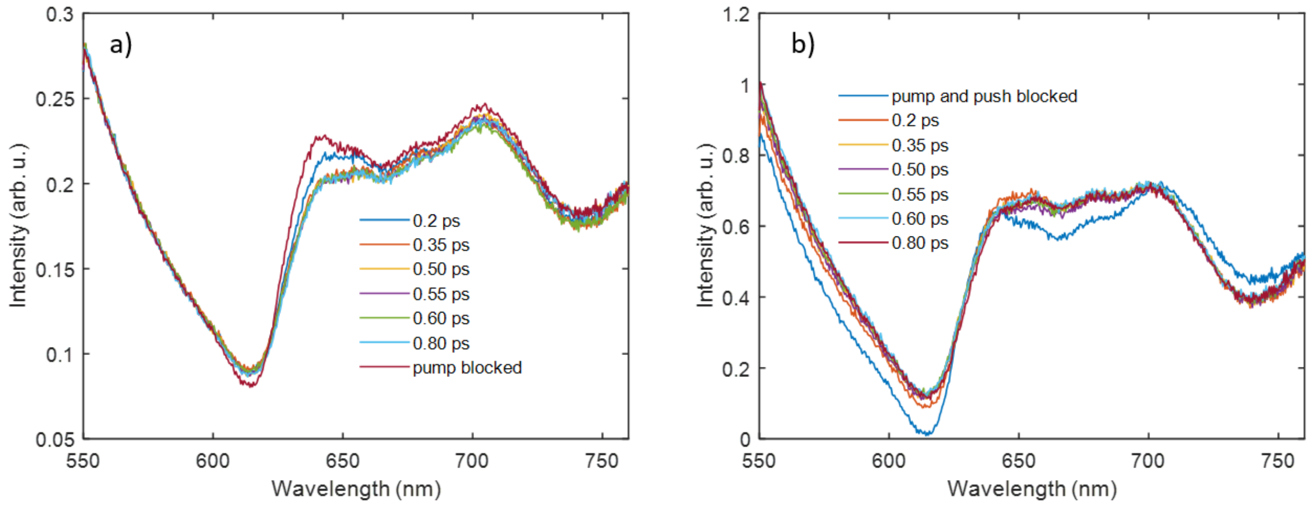

Figure S1. Transmission spectra of WS_2/Au in PP and PPP configurations at different pump-probe delays t_2. a) PP: pump pulse centered at 515 nm (2.4 eV) with a fluence of 200 uJ/cm^2. b) PPP: pump pulse centered at 1030 nm (1.2 eV) with a fluence of 1.7 mJ/cm2 and push pulse centered at 515 nm (2.4 eV) with a fluence of 200 uJ/cm^2.

Supplementary Note 2: PP 1030 nm on WS_2/Au

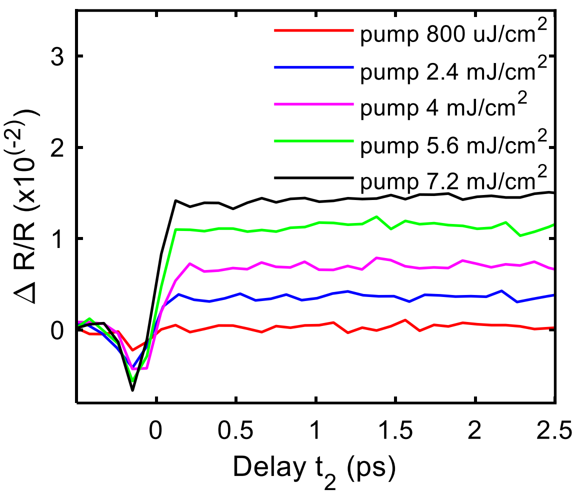

Figure S2. Pump fluence dependence at λ_{signal} = 610 nm (2.03 eV) on WS_2/Au in PP configuration with pump pulse centered at 1030 nm.

Supplementary Note 3: Substrate roughness

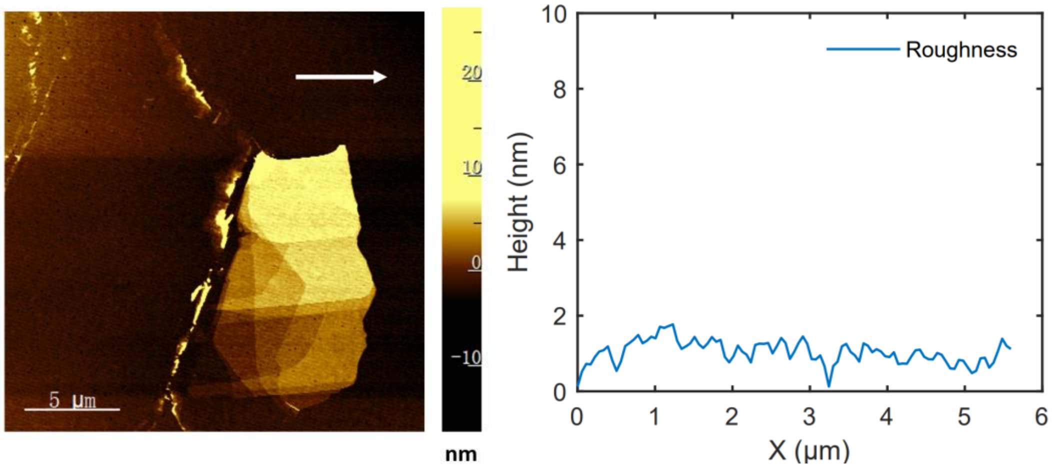

Figure S3. AFM measurement on the gold substrate.

Supplementary Note 4: Experiments

We generated the second harmonic of a Yb:KGW amplified laser, operating at 50 kHz repetition rate, to produce pulses centered at 515 nm (2.4 eV), with a duration of 150 fs as a pump(push) pulse in the PP(PPP) measurements. The pulse duration of the fundamental (pump pulse in the PPP measurements) at 1030 nm (1.2 eV) was 220 fs. As a probe pulse we used visible white light generated by the

fundamental laser pulses in a YAG crystal. Due to the narrowband detection, temporal compression of the probe pulse is not necessary. The temporal overlap between pump and probe $t_2 = 0$ is defined as the time when the normalized signal is equal to 0.5. The temporal overlap between pump and push $t_1 = 0$ was determined by generating a nonlinear optical signal between the two pulses. Modulation of the exciting pulse (515 nm) was achieved with a pockel cell. The sensitivity of our pump-probe setup allows to detect a variation of the transient signal given a root-mean-square of the noise floor on the order 10^{-4} to 10^{-5}.

Supplementary Note 5: Sample fabrication

To ensure a flat surface of the gold back reflector rather than the rough surface of the evaporated Au, an epoxy-based peeling procedure was applied to the 100 nm-thick Au film evaporated on a clean polished Si wafer (parent wafer) using an e-beam evaporator (Kurt J. Lesker PVD 75). A piece of silicon wafer (transfer wafer) was glued to the Au film using a thin layer of thermal epoxy (Epo-Tek 375, Epoxy Technology) and then peeled upwards after the epoxy layer achieves its final hardness (curing), resulting in stripping of the Au film from the parent wafer. WS_2 was mechanically exfoliated from bulk crystal (HQ-graphene) using Scotch Tape and transferred onto the Au substrate.

Supplementary Note 6: Fit model

All time constants for the fast decay of the transient signals have been extracted by fitting a single-exponential model:

$$y(t_2) = a \cdot \exp\left((-1) \cdot \left(\frac{t_2 - t_0}{\tau}\right)\right) + G.$$

The fast decay of the transient signals is fitted considering only pump-probe delays t_2 larger than 0.2 ps until t_2 = 2.5 ps.
